# *SREBF1* gene variations modulate insulin sensitivity in response to a fish oil supplementation

**DOI:** 10.1186/1476-511X-13-152

**Published:** 2014-10-01

**Authors:** Annie Bouchard-Mercier, Iwona Rudkowska, Simone Lemieux, Patrick Couture, Louis Pérusse, Marie-Claude Vohl

**Affiliations:** Institute of Nutrition and Functional Foods (INAF), Laval University, 2440 Hochelaga Blvd., Quebec, G1V 0A6 Canada; Department of Food Science and Nutrition, Laval University, 2425 de l’Agriculture St., Quebec, G1K 7P4 Canada; Endocrinology and Nephrology, CHU de Québec Research Center, 2705 Laurier Blvd., Quebec, G1V 4G2 Canada; Department of Kinesiology, Laval University, Quebec, G1K 7P4 Canada

**Keywords:** *SREBF1* gene, Fish oil supplementation, Insulin sensitivity, Inter-individual variability, Polymorphisms

## Abstract

**Background:**

An important inter-individual variability in the response of insulin sensitivity following a fish oil supplementation has been observed. The objective was to examine the associations between single nucleotide polymorphisms (SNPs) within *sterol regulatory element binding transcription factor 1* (*SREBF1*) gene and the response of insulin sensitivity to a fish oil supplementation.

**Methods:**

Participants (n = 210) were recruited in the greater Quebec City area and followed a 6-week fish oil supplementation protocol (5 g/day: 1.9-2.2 g EPA; 1.1 g DHA). Insulin sensitivity was assessed by the quantitative insulin sensitivity check index (QUICKI). Three tag SNPs (tSNPs) within *SREBF1* gene were genotyped according to TAQMAN methodology.

**Results:**

Three tSNPs (rs12953299, rs4925118 and rs4925115) covered 100% of the known genetic variability within *SREBF1* gene. None of the three tSNPs was associated with either baseline fasting insulin concentrations (rs12953299, rs4925118 and rs4925115) (p = 0.29, p = 0.20 and p = 0.70, respectively) or QUICKI (p = 0.20, p = 0.18 and p = 0.76, respectively). The three tSNPs (rs12953299, rs4925118 and rs4925115) were associated with differences in the response of plasma insulin levels (p = 0.01, p = 0.005 and p = 0.004, respectively) and rs12953299 as well as rs4925115 were associated with the insulin sensitivity response (p = 0.009 and p = 0.01, respectively) to the fish oil supplementation, independently of the effects of age, sex and BMI.

**Conclusions:**

The genetic variability within *SREBF1* gene has an impact on the insulin sensitivity in response to a fish oil supplementation.

**Trial registration:**

clinicaltrials.gov: NCT01343342.

## Introduction

The *sterol regulatory element binding transcription factor 1* (*SREBF1*) gene encodes a transcription factor which is a main regulator of lipid metabolism, the sterol regulatory element-binding protein-1c (SREBP-1c) [1]. *SREBF1* gene is expressed in multiple tissues including liver, white and brown adipose tissue, adrenal gland and to a lower extent in pancreatic β-cell [2, 3]. Insulin induces the expression of the *SREBF1* gene in adipose tissue, liver and muscle cells [1]. However, in pancreatic β-cell, it has been observed that SREBP-1c modulates insulin secretion potentially through a mechanism involving lipotoxicity [3, 4]. SREBP-1c may be involved in reticulum endoplasmic stress and in β-cell apoptosis [5]. The knockdown of SREBP-1c in pancreatic β-cell inhibited the expression of markers of reticulum endoplasmic stress [5].

*SREBF1* gene expression is also regulated by dietary intakes. For example, an insulin independent effect has been demonstrated with different types of sugar such as glucose, fructose and sucrose, on *SREBF1* gene expression induction [6]. Dietary fats also affect *SREBF1* gene expression; a high saturated fat (SFA) diet increases *SREBF1* gene expression both in the liver and in pancreatic β-cell [3, 7] whereas a diet high in polyunsaturated fat (PUFA) decreases *SREBF1* gene expression [3, 8, 9]. The intake of fish oil may have a favorable impact on insulin sensitivity. Among fructose-induced hypertriglyceridemic and insulin resistant male rhesus macaques, the intake of 4 g/day of fish oil prevented the development of hypertriglyceridemia and insulin resistance [10]. Studies observing rodents have also observed a beneficial effect of fish oil on insulin sensitivity [11, 12]. In diet induced obese mice, the intake of fish oil reduces *SREBF1* gene expression levels in the liver and modifies the expression of other genes involved in lipid metabolism such as *fatty acid synthase* gene (*FASN*) and *acyl-Coenzyme A oxidase 1* (*ACOX1*) [13]. Eicosapentaenoic acid (EPA) was shown to inhibit SREBP-1 maturation [14] and to restore insulin secretion after suppression by palmitate through an SREBP-1c dependent mechanism [15].

The genetic variability within the *SREBF1* gene may play a role in insulin resistance or type 2 diabetes. A meta-analysis of genome wide scans in European populations showed linkage with type 2 diabetes in the 17p11 region, which comprises the *SREBF1* gene [16]. Single nucleotide polymorphisms (SNPs) within the *SREBF1* gene have been associated with type 2 diabetes, insulin resistance, obesity and blood lipid levels [17–22]. Among humans the impacts of fish oil on insulin sensitivity, glucose concentrations and/or the risk of type 2 diabetes have not been consistent [23–27]. Some studies even observed an increase in fasting insulin concentrations and/or fasting glucose concentrations [28, 29]. These inconsistencies in results could be partly due to differences in the genetic background, dietary intakes and/or lifestyle. Our group previously observed a large inter-individual variability in the response of insulin sensitivity to a fish oil supplementation [30]. Thus, the objective of this study was to examine the associations between SNPs within *SREBF1* gene and the plasma insulin and glucose response to a fish oil supplementation.

## Methods

### Participants

Methods related to this study cohort have been previously described [31]. Briefly, a total of 254 unrelated participants from the greater Quebec City metropolitan area were recruited to participate in this clinical trial between September 2009 and December 2011 through advertisements in local newspapers as well as by electronic messages sent to university students/employees. To be eligible, participants had to be non-smokers and without any thyroid or metabolic disorders requiring treatment, for example diabetes, hypertension, severe dyslipidemia, and/or coronary heart disease. A total of 210 participants completed the fish oil supplementation period. However, fasting insulin and glucose concentrations were obtained only for 207 participants. The experimental protocol was approved by the ethics committees of Laval University Hospital Research Center and Laval University. This clinical trial was registered at clinicaltrials.gov (NCT01343342). Informed written consent was obtained from all the study participants.

### Study design and diets

The study design and diets have been described previously [31]. Briefly, participants followed a run-in period of 2 weeks. Individual dietary instructions were given by a trained dietitian to achieve the recommendations from Canada’s Food Guide. After the 2-week run-in period, each participant received a bottle containing fish oil capsules for the next 6 weeks. They were instructed to take five capsules (1 g of fish oil/capsule) per day (Ocean Nutrition, Nova Scotia, Canada), providing a total of 5 g of fish oil (1.9-2.2 g EPA and 1.1 g docosahexaenoic acid (DHA)) per day. Compliance was assessed from the return of bottles and by measuring erythrocyte membranes and plasma phospholipids fatty acid (FA) composition. Dietary intakes were assessed at screening using a validated food frequency questionnaire (FFQ) [32]. Dietary intakes were also measured pre- and post-supplementation using two 3-day dietary records.

### Biochemical parameters

The morning after a 12-hour overnight fast and 48-h alcohol abstinence, blood samples were collected from an antecubital vein into vacutainer tubes containing EDTA. Blood samples were collected at screening, baseline, pre-supplementation (two weeks after baseline) and post-supplementation (six weeks after pre-supplementation). Plasma was separated by centrifugation (2500 × g for 10 minutes at 4°C), samples were aliquoted and frozen for subsequent analyses. Plasma total cholesterol (total-C) and plasma triglyceride concentrations were measured using enzymatic assays [33, 34]. Infranatant (d >1.006 g/ml) with heparin-manganese chloride was used to precipitate VLDL and LDL and then determine HDL-cholesterol concentrations (HDL-C) [35]. The equation of Friedewald was used to estimate LDL-cholesterol concentrations (LDL-C) [36]. Non-HDL-C was calculated by subtracting HDL-C from total-C. Fasting insulin concentrations were measured by radioimmunoassay with polyethylene glycol separation [37]. Fasting glucose concentrations were enzymatically measured [38]. The quantitative insulin sensitivity check index (QUICKI) was used as a marker of insulin sensitivity calculated as follow: 1/(log(insulin(mU/L)) + log(glucose (mg/dL))) [39]. QUICKI has been reported to be more reproducible than the common homeostasis model assessment (HOMA) of insulin resistance [40] and has a strong linear correlation with glucose clamp estimates among various types of health conditions (healthy, obesity, insulin resistance, diabetes and hypertension) [41].

### SNPs selection and genotyping

**Figure 1:**
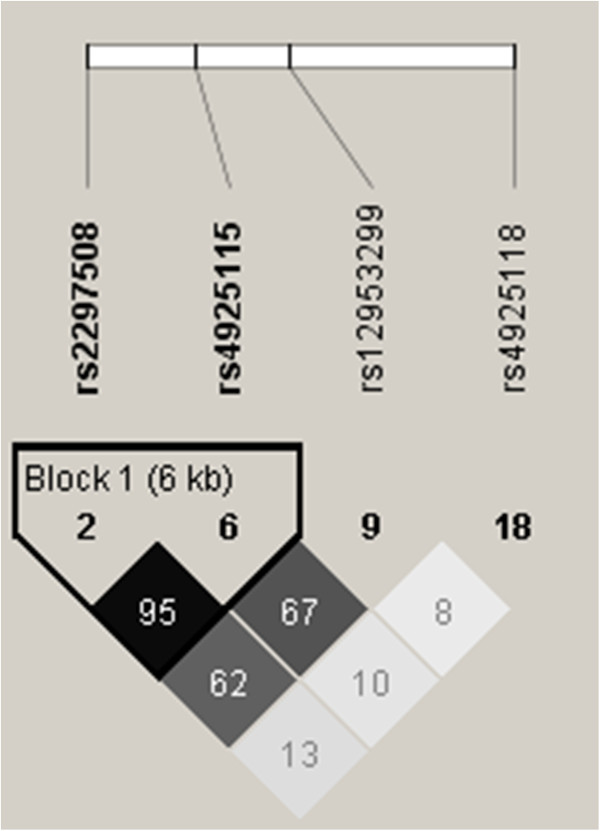
**Linkage disequilibrium (LD) plot of tSNPs within** ***SREBF1*** **gene.** Figure legend. LD plots were generated by HaploView software version 4.2 using r^2^ LD values. Two of the selected tSNPs (rs12953299 and rs4925115 of the first cohort) were in moderate to high LD with the exonic SNP rs2297508 (second cohort) (rs12953299 (r^2^ = 0.62) and rs4925115 (r^2^ = 0.95)).

As described previously [31], SNPs were selected with the International HapMap Project SNP database (HapMap Data Rel 28 Phase II + III, August 10, on National Center for Biotechnology Information (NCBI) B36 assembly, dbSNP b126). Tag SNPs (tSNPs) were determined with the tagger procedure in HaploView software version 4.2 with minor allele frequency (MAF) of >0.05 and pairwise tagging r^2^ ≥ 0.80. Afterwards, as shown in Figure 1, linkage disequilibrium (LD) plot were generated with Haploview software version 4.2. Figure 1 also illustrates the high LD between the chosen tSNP rs4925115 and the well described rs2297508 located in exon 18c of *SREBF1* gene [17, 20, 21]. All tSNPs were genotyped within INAF laboratories with the TAQMAN methodology [42], as described previously [43]. Briefly, genotypes were determined using ABI Prism SDS version 2.0.5 (Applied Biosystem, Foster City, CA, USA). All SNPs were successfully genotyped.

### Fatty acid composition of erythrocyte membranes and plasma phospholipids

As described previously [44], FA composition was measured in erythrocyte membranes by gas chromatographic analysis in a subset of 31 participants. Methods to extract plasma phospholipids have been described elsewhere [31]. FA composition from plasma phospholipids were measured on the total cohort of 210 participants. Briefly, plasma lipids were extracted according to a modified Folch method [45]. Capillary gas chromatography was used to obtain FA profiles [46]. FA profiles both in erythrocyte membranes and plasma phospholipids were expressed as the relative percentage areas of total FAs.

### Gene expression assessment

Blood samples (pre- and post- supplementation) were collected into an 8-ml Cell Preparation Tube (CPT) (Becton Dickinson, Oakville, On, Canada). Gene expression levels were measured in peripheral blood mononuclear cells (PBMCs), which are considered a valid proxy measure for many tissues including the liver [47, 48]. Peripheral blood mononuclear cells (PBMCs) were separated by centrifugation (1500 × g, 20 min, at room temperature) and washed according to the manufacturer’s instructions. Total RNA was extracted with RNeasy Plus Mini Kit (QIAGEN, Mississauga, On, Canada) according to manufacturer’s protocol. Spectrophotometric quantification was realised with NanoDrop 2000C UV–vis Spectrophotometer (Thermo Scientific) and cDNA was generated using 400 ng of total RNA with the High Capacity cDNA Reverse Transcription Kit (Life Technologies™). cDNA was mixed with TaqMan OpenArray® Real-Time PCR Master Mix (#4462164, Life Technologies™). The assays used were as follows: Hs01088691_m1 (*SREBF1*) and *GAPDH* Hs99999905_m1 as the housekeeping gene. All assays used the same fluorescent reporter probe (FAM dye labeled). All samples were run in triplicate on a QuantStudio™ 12 K Flex Real-Time PCR (RT-PCR) System (Life Technologies™) using 48-well plates TaqMan® OpenArray® RT PCR Inventoried Format 18. The RT-PCR results were analysed with ExpressionSuite software v1.0.1 (Life Technologies™).

### Second cohort

Seven hundred (700) Caucasians aged between 18 and 55 years were recruited in the Quebec City metropolitan region. Recruitment occurred between 2004 and 2006 through public advertisements (local newspapers and electronic messages) sent to university and hospital employees, as described previously [49]. A trained research assistant took anthropometric measures. A registered dietitian administered a validated FFQ to assess dietary intakes [32]. *SREBF1* c.*619C > G (rs2297508) was genotyped using the TAQMAN methodology [42]. Statistical analyses were performed by using a model including the interaction term SNP*PUFA with the GLM procedure in SAS and the type 3 sum of squares for unbalanced study design. Age, sex, BMI and total energy intakes were considered as confounding variables.

### Statistical analyses

The Hardy-Weinberg equilibrium was tested with the ALLELE procedure of SAS version 9.3 using Fisher’s exact test (P < 0.01). When the genotype frequency for homozygous individuals of the minor allele was <5%, carriers (heterozygotes and homozygotes) of the minor allele were grouped.

The sample size was calculated based on plasma triglyceride changes following the fish oil supplementation with a genetic variation occurring in a relatively low frequency (5%) of the population. A group of 152 participants was sufficient to provide an 80% probability and a 5% significance level of detecting an anticipated difference of 0.25 mmol/L in plasma triglyceride concentrations after 6 weeks of fish oil supplementation.

Non-normally distributed variables were logarithmically transformed. Fasting insulin concentration values higher or lower than means ± 3 multiplied by standard deviation (SD) were considered as outliers (n = 6), thus 201 participants were kept for the statistical analyses. Differences were assessed using analyses of variance (ANOVA) with the GLM procedure in SAS and the type 3 sum of squares for unbalanced study design. The fasting insulin response (delta) was calculated as followed: ((post-supplementation insulin concentrations minus pre-supplementation insulin concentrations)/pre-supplementation insulin concentrations*100). The same model was used to test the associations with fasting glucose concentrations, insulin sensitivity (QUICKI) and FA composition both in erythrocytes and plasma phospholipid membranes. Each model was adjusted for the effects of age, sex and BMI. To take into account the impact of multiple testing, the simpleM method described by Gao *et al*. [50] was utilised. Briefly, this method considers the impacts of LD between SNPs and has been demonstrated as efficient and accurate comparatively to permutation-based corrections [50]. First, the composite LD correlation matrix was derived from the data set. Then, eigenvalues were calculated using the SAS PRINCOMP procedure and the number of effective independent tests was inferred so that the corresponding eigenvalues explain 99.5% of the variation in SNP data or the variables (fasting glucose, insulin and QUICKI), as proposed by Gao *et al*. [50]. According to Gao’s method, the number of effective independent tests for the three SNPs was 2 and for the three traits (fasting glucose, insulin and QUICKI) was 2. The final step applies the Bonferroni correction formula to calculate the adjusted point-wise significance level, which was defined as α_G_ = 0.05/(2X2) (effective independent tests). Thus, p-values <0.0125 were considered significant (p = 0.05/(4)). All statistical analyses were performed using SAS statistical software version 9.3 (SAS Institute, Inc., Cary, NC, USA).

## Results

All tSNPs were in Hardy-Weinberg equilibrium. Three tSNPs covered 100% of the known genetic variability within the *SREBF1* gene [31]. As presented in Figure 1, two of the selected tSNPs were in moderate to high LD with the exonic SNP rs2297508 (rs12953299 (r^2^ = 0.62) and rs4925115 (r^2^ = 0.95)).

Baseline characteristics of the study participants are shown in Table 1. Before the fish oil supplementation period, no differences in fasting insulin concentrations according to genotypes were observed for the three tSNPs (rs12953299, rs4925118 and rs4925115) (p = 0.29, p = 0.20 and p = 0.70, respectively). Also there were no differences in either fasting glucose concentrations according to genotypes of rs12953299, rs4925118 and rs4925115 (p = 0.16, p = 0.64 and p = 0.22, respectively) or for insulin sensitivity (QUICKI) values (p = 0.20, p = 0.18 and p = 0.76, respectively).

**Table 1:** **Descriptive characteristics at baseline, pre-supplementation and post-supplementation (n = 201)**

Variables	Means ± SD	Pre-supplementation	Post-supplementation	P-value ^1^
**Age (years)**	30.9 ± 8.7	-	-	
**Sex (men/women)**	92/109	-	-	
**BMI (Kg/m** ^**2**^ **)**	27.6 ± 3.5	27.6 ± 3.5	27.7 ± 3.6	0.03
**Waist circumference (cm)**	Men: 94.5 ± 10.5	Men: 94.4 ± 10.3	Men: 94.8 ± 10.3	0.10
	Women: 91.5 ± 10.2	Women: 91.5 ± 9.9	Women: 91.4 ± 10.2	0.65
**Fasting glucose (mmol/L)**	4.94 ± 0.53	4.95 ± 0.44	5.04 ± 0.49	0.0002
**Fasting insulin (pmol/L)**	79.0 ± 27.5 (n = 199)	77.7 ± 29.3	79.0 ± 30.0	0.52
**QUICKI**	0.367 ± 0.018 (n = 199)	0.338 ± 0.019	0.336 ± 0.020	0.19
**HOMA-IR**	2.51 ± 1.00 (n = 199)	2.48 ± 1.01	2.57 ± 1.06	0.12
**Total-C (mmol/L)**	4.80 ± 1.01	4.74 ± 0.90	4.71 ± 0.95	0.45
**LDL-C (mmol/L)**	2.78 ± 0.87	2.75 ± 0.81	2.77 ± 0.86	0.40
**HDL-C (mmol/L)**	Men : 1.29 ± 0.31	Men : 1.29 ± 0.30	Men : 1.29 ± 0.33	0.72
	Women : 1.61 ± 0.40	Women : 1.58 ± 0.36	Women : 1.64 ± 0.40	0.002
**Triglycerides (mmol/L)**	1.21 ± 0.60	1.19 ± 0.60	1.00 ± 0.46	<0.0001

Means ± SD.

^1^P-values were determined using a paired t-test and compared post-supplementation to pre-supplementation values.

QUICKI: quantitative insulin sensitivity check index; HOMA-IR: homeostasis model assessment insulin resistance.

Globally, during the study protocol fasting insulin concentrations were not modified (pre-supplementation insulin concentrations: 77.7 ± 29.3pmol/L; post-supplementation insulin concentrations: 79.0 ± 30.0pmol/L) (p = 0.52)). Fasting glucose concentrations slightly increased from 4.95 ± 0.44 mmol/L to 5.04 ± 0.49 mmol/L after the fish oil supplementation period (p = 0.0002), as previously reported [51]. An important inter-individual variability has been observed in the response of fasting insulin concentrations, ranging from a decrease of -53.0% to an increase of +135.2%. Briefly, 110 individuals decreased (relative change ≤ 0%) and 91 increased (relative change > 0%) their fasting insulin concentrations. Globally, the mean change in insulin concentrations was 5.1% ± 30.0%. The insulin sensitivity (QUICKI) was not modified by the fish oil supplementation (p = 0.19).

No differences according to genotypes of the *SREBF1* gene were observed in the response of fatty acid n-3 PUFA (EPA, DHA and total n-3 PUFA) phospholipid content to the fish oil supplementation. A difference was observed for EPA concentrations in erythrocyte membranes according to rs4925118 genotypes (p = 0.02) for which the relative increase following the fish oil supplementation was greater among C/C homozygotes than for the T allele carriers (T/T + C/T = 147.7 ± 80.0% (n = 4); C/C = 236.5 ± 73.0% (n = 23)). A trend (p = 0.07) was also observed for pre-supplementation EPA content in erythrocyte membranes according to rs4925118 genotypes (T/T + C/T = 0.88 ± 0.33% (n = 4); C/C = 0.68 ± 0.22% (n = 24)).

*SREBF1* gene expression levels were not modified by the fish oil supplementation (p = 0.85). As shown in Table 2, no differences were observed in the response of *SREBF1* gene expression levels to fish oil supplementation between genotypes of rs4925118 and rs4925115 (p = 0.59, p = 0.47 and p = 0.25, respectively).

**Table 2:** **Gene expression response according to genotypes of SNPs within** ***SREBF1*** **gene**

SNPs	Genotype	Fold change ^1^	P-value ^2^
rs12953299	A/A (n = 45)	1.04 ± 0.36	0.59
	A/G (n = 98)	1.05 ± 0.27	
	G/G (n = 55)	1.00 ± 0.22	
rs4925118	T/T + C/T (n = 66)	1.06 ± 0.26	0.47
	C/C (n = 132)	1.02 ± 0.29	
rs4925115	A/A (n = 32)	1.00 ± 0.25	0.25
	A/G (n = 103)	1.07 ± 0.32	
	G/G (n = 63)	1.00 ± 0.23	

Means ± SD.

^1^The fold change represents post-supplementation relative gene expression levels compared to pre-supplementation relative gene expression levels.

Fold change = 2^-∆∆CT^ = 2^-(post-supplementation ∆CT-pre-supplementation ∆CT)^.

^2^P*-*values were calculated with an ANOVA adjusted for age, sex and BMI.

SNPs: single-nucleotide polymorphisms; *SREBF1*: *sterol regulatory element binding transcription factor 1.*

**Figure 2:**
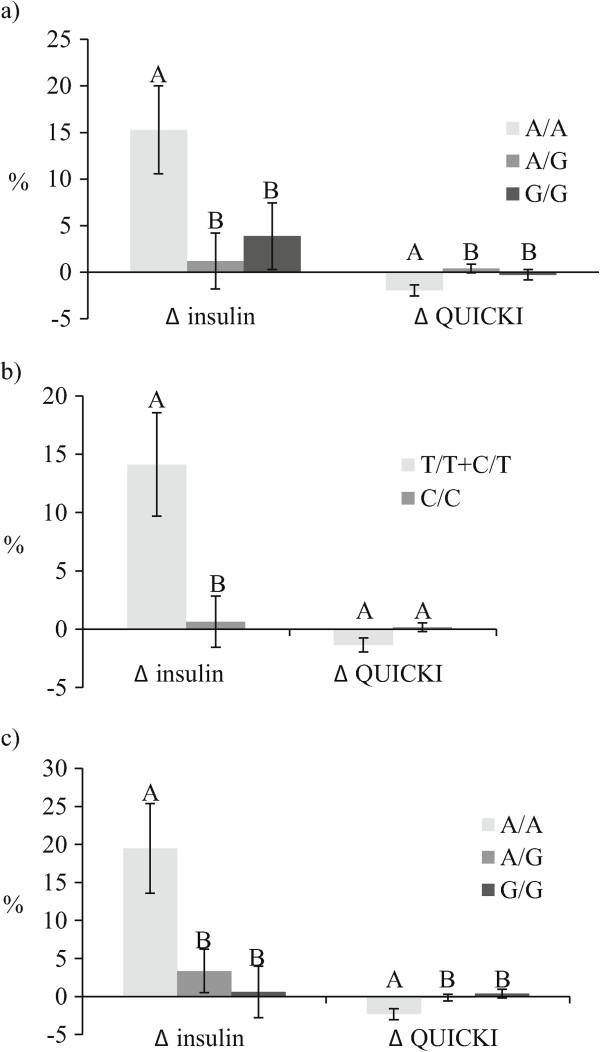
**The relative response in fasting insulin concentrations and QUICKI index (insulin sensitivity) according to genotype.** Figure legend. **a)** rs12953299 (A/A: n = 46, A/G: n = 100, G/G: n = 55); Delta insulin (A/A: 15.3 ± 32.0%, A/G: 1.2 ± 30.1%, G/G: 3.9 ± 26.4%), P-value for delta insulin model: p = 0.01; Delta QUICKI (A/A: -2.0 ± 4.1%, A/G: 0.4 ± 4.8%, G/G: 3.9 ± 26.4%), P-value for delta QUICKI model: p = 0.009 **b)** rs4925118 (T/T + C/T: n = 67, C/C: n = 134); Delta insulin (T/T + C/T: 14.1 ± 36.2%, C/C: 0.6 ± 25.3%), P-value for delta insulin model: p = 0.005; P-value for delta QUICKI model: p = 0.16 **c)** rs4925115 (A/A: n = 33, A/G: n = 105, G/G: n = 63); Delta insulin (A/A: 19.5 ± 34.0%, A/G: 3.4 ± 29.3%, G/G: 0.6 ± 27.0%), P-value for delta insulin model: p = 0.004; Delta QUICKI (A/A: -2.3 ± 4.2%, A/G: -0.1 ± 4.5%, 0.4 ± 4.5%), P-value for delta QUICKI model: p = 0.01. Delta values (relative change) were calculated as ((post-supplementation values minus pre-supplementation values)/pre-supplementation values*100). All differences were assessed with ANOVA adjusted for age, sex and BMI. Means ± SE.

As shown in Figure 2, the% change of fasting insulin concentrations ((post-supplementation insulin concentrations minus pre-supplementation insulin concentrations)/pre-supplementation insulin concentrations*100) following the fish oil supplementation was different according to genotypes of the three tSNPs (rs12953299, rs4925118 and rs4925115) within the *SREBF1* gene, adjusted for age, sex and BMI (p = 0.01, p = 0.005 and p = 0.004, respectively). The response of fasting glucose concentrations was not different according to genotypes of the tSNPs (rs12953299, rs4925118 and rs4925115). The insulin sensitivity (QUICKI) was associated with the tSNPs rs12953299 and rs4925115 (p = 0.009 and p = 0.01, respectively) but not with rs4925118 (p = 0.16). The potential effects of total dietary n-3 PUFA intakes in the participants’ habitual diets were included as a confounding variable in these models and did not modify the associations observed (data not shown).

### Second cohort

**Figure 3:**
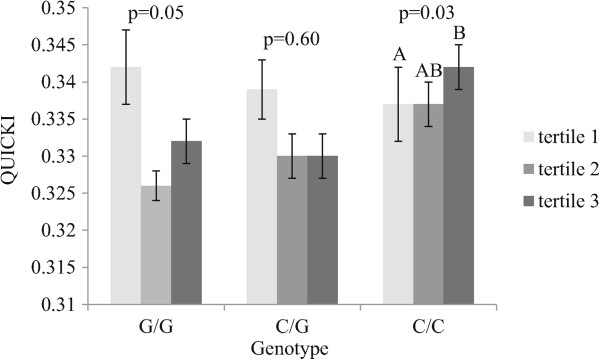
**QUICKI index values according to rs2297508 genotype and tertiles of dietary PUFA intakes.** Figure legend. Tertile 1 of dietary PUFA intakes (3.17 g-11.97 g) (G/G: n = 48; C/G: n = 95; C/C: n = 67), Tertile 2 of dietary PUFA intakes (11.98 g-16.49 g) (G/G: n = 41; C/G: n = 91; C/C: n = 82) and Tertile 3 of dietary PUFA intakes (16.53 g-48.18 g) (G/G: n = 27; C/G: n = 96; C/C: n = 86). Differences in QUICKI values between tertiles were assessed with an ANOVA by genotype adjusted for the effects of age, sex and BMI. Means with different letters are significantly different. Means ± SE.

As presented in Table 3, one significant gene-diet interaction effects on QUICKI (insulin sensitivity index) was observed between rs2297508 and dietary PUFA intakes (in grams) (p = 0.05). To further understand these associations dietary PUFA intakes were divided in tertiles. Figure 3 presents QUICKI values according to genotype of rs2297508 and tertiles of dietary PUFA intakes. A significant difference was observed only among C/C homozygotes for which individuals with the highest dietary PUFA intakes had higher QUICKI values than individuals with the lowest dietary PUFA intakes (p = 0.03). A trend (p = 0.06) was observed for the interaction effect on QUICKI between rs2297508 and dietary intakes of omega-3 PUFA (in grams). Both models were adjusted for the effects of age, sex, BMI and energy intakes. Genotype of rs2297508 alone was not associated with QUICKI values (p = 0.21). No gene-diet interaction effects were observed on fasting insulin or glucose concentrations.

**Table 3:** **Gene-diet interaction effects on QUICKI between rs2297508 and PUFA intakes (total and n-3 PUFA)**

	Dietary n-3 PUFA intake (in grams)		Dietary PUFA intake (in grams)	
Genotype	β (Interaction term)	P Interaction effect ^1^	β (Interaction term)	P Interaction effect ^1^
G/G (n = 125)	-0.0194 ± 0.0083	0.06	-0.0163 ± 0.0067	0.05
C/G (n = 297)	-0.0073 ± 0.0064		-0.0073 ± 0.0052	
C/C (n = 242)	0		0	

Means ± SD.

^1^ANOVA adjusted for age, sex, BMI and energy intakes considering the interaction effect between genotype and dietary fat intakes.

PUFA: polyunsaturated fatty acid.

## Discussion

Participants of this cohort were overweight but generally considered as healthy according to lipid concentration values [52]. Fasting glucose concentrations were within normal values [53]. The QUICKI index indicated a probable borderline insulin resistance state among these participants [39, 41, 54]. An important inter-individual variability in the response of fasting insulin concentrations was observed. This wide inter-individual variability in HOMA-insulin sensitivity (IS) response in this cohort has been previously described [30]. In this study, effects of tSNPs within the *SREBF1* gene on the fasting insulin and insulin sensitivity responses were observed after the fish oil supplementation.

*SREBF1* gene is an important transcription factor regulating many genes involved in lipid metabolism and also in insulin induced glucose metabolism [1]. Moreover, the expression of *SREBF1* gene is significantly affected by dietary intakes, including fish oil [3, 6–9, 13]. Therefore, *SREBF1* gene is an interesting candidate for the study of inter-individual variability in the response of fasting insulin concentrations to a fish oil supplementation. Whether these impacts are mediated through the effects of SREBP-1c within hepatocytes and/or pancreatic cells is unknown. In the liver, increased SREBP-1c concentrations have been shown to repress the transcription of *insulin receptor substrate 2* (*IRS2*) gene which led to a detrimental impact on insulin sensitivity, a fatty liver and a production of VLDL enriched in triglycerides [55]. IRS2 mediates insulin signaling in the liver [55]. Insulin in the liver activates glycogen synthesis, inhibits hepatic glucose output and promotes lipogenesis [55]. The induction of SREBP-1c in mice resulted in impaired secretion and glucose intolerance, as reviewed by Shimano *et al*. [3]. SREBP-1c may also affect insulin secretion of pancreatic β-cell through a mechanism involving uncoupling protein-2 (UCP2) [56]. A sterol regulatory element (SRE) has been discovered in the promoter region of *UCP2* gene [56]. An increase in *UCP2* gene expression is associated with a lower efficacy of glucose-induced insulin secretion [57]. In the present study no differences in *SREBF1* gene expression were observed following the fish oil supplementation. Studies examining the impacts of PUFA, fish oil or EPA on *SREBF1* gene regulation have been conducted among mice or *in vitro* with human cells [6–9, 13–15]. It is possible that the dose used for the supplementation in this study was insufficient to observe an effect on *SREBF1* gene expression. However, the activity of lipogenic target genes of SREBP-1c transcription factor is not only regulated by *SREBF1* mRNA abundance. For example, Tanaka *et al*. [14] did not observe reduced *SREBF1* mRNA levels but rather an inhibition of the maturation of SREBP-1c. Thus, the fish oil supplementation in the present study may have had effects on the insulin response through posttranslational modifications of SREBP-1c and its impact on subsequent target genes. However, we cannot rule out the possibility that significant differences may have been observed if expression levels were directly measured in hepatocytes [58].

The overall effects of fish oil intakes on insulin resistance, glycemic control and the risk of type 2 diabetes appears to be negligible, as recently reviewed by Wu *et al*. [59]. However, the authors observed a large heterogeneity. Thus, it is possible that for some individuals the impacts of fish oil intake on the risk of type 2 diabetes or other related biologic parameters may be beneficial and for some other individuals detrimental. Quite a few studies have observed a modest increase in fasting glucose concentrations after fish oil intake [60]. It has been observed that the reduction in plasma triglyceride concentrations induced by n-3 PUFA intake may be partly induced by the increased use of glycerol for gluconeogenesis which may explain increases in fasting glucose concentrations [61, 62]. The increase in fasting glucose concentrations was also observed in this cohort [51]. However, because this study was designed without a control group, we cannot rule out the possibility that part of these changes may be related to modifications in the participants’ lifestyle during the protocol. Still, participants were asked to maintain their nutritional and physical activity habits stable during the intervention. Assessment of dietary intakes and physical activity levels during the study protocol revealed that they were quite stable (data not shown).

In the present study, homozygotes for minor alleles of the tSNPs rs12953299 and rs4925115 were associated with an increase in fasting insulin concentrations and a decrease in insulin sensitivity assessed by QUICKI after a fish oil supplementation compared to the other genotypes. For the tSNP rs4925118 only a difference in the fasting insulin response was observed, carriers of the T allele increased their fasting insulin concentrations after a fish oil supplementation compared to C/C homozygotes. Thus, for these genotypes the impact of fish oil on insulin sensitivity may be detrimental. For the other genotypes, the fish oil supplementation had a minor impact which may be less likely to increase the risk of type 2 diabetes. The genetic variability within transcription factors such as the *SREBF1* gene, which are affected by fish oil intake may be the key to understanding the variability observed in the response of fasting insulin concentrations and insulin sensitivity [63]. SNPs within the *SREBF1* gene have been frequently associated with type 2 diabetes or insulin resistance. A meta-analysis of four European genome screens found the strongest linkage with type 2 diabetes on chromosome 17p11.2-q22 where is located the *SREBF1* gene [16]. One SNP (rs2297508) within *SREBF1* gene has been reported across a few populations to be associated with the risk of type 2 diabetes [17, 18, 20, 21]. Felder *et al*. [21] found that the C/G and the G/G genotypes of rs2297508 had a ~1.4-fold higher risk of type 2 diabetes. In a French cohort, rs2297508 was also associated with obesity and type 2 diabetes independently of the obesity status [17]. Moreover, the SNP rs2297508 was related to sex-specific differences in the response of lipid and insulin concentrations as well as in HOMA-IR to a diet high in carbohydrates [64]. In their study, Zhang et al. [64] observed that the C allele of rs2297508 was associated with more favorable impacts on plasma triglyceride, fasting insulin and HOMA-IR than the G allele. In our study, the genotype alone was not associated with QUICKI values. However, a gene-diet interaction effect between rs2297508 and dietary PUFA intakes was observed. A large-scale gene-centric meta-analysis identified the *SREBF1* gene as a type 2 diabetes loci among Europeans, with rs4925115 being the most significantly associated SNP [19]. The three tSNPs (rs12953299, rs4925115 and rs4925118) covered 100% of the known genetic variability within the *SREBF1* gene. However, two of these tSNPs were in moderate LD (rs12953299 and rs4925115, r^2^ = 0.68) and were also in moderate to high LD with the most studied *SREBF1* gene SNP rs2297508 (r^2^ = 0.62 and r^2^ = 0.95, respectively). Since SNPs within the *SREBF1* gene including the promoter region, are in moderate to high LD, we cannot rule out the possibility that the tSNPs examined herein may also be in LD with SNPs within the *SREBF1* gene promoter region which could affect its expression [65]. In the second cohort, the SNP rs2297508 from *SREBF1* gene interacted with dietary PUFA intakes to affect insulin sensitivity (assessed by QUICKI). This SNP is located in exon 18c and is synonymous (Gly952Gly). Thus, these results may indicate that the genetic variability within the *SREBF1* gene has an impact on the response of insulin sensitivity to n-3 PUFA and/or PUFA intakes.

## Conclusion

To our knowledge this study was the first to examine associations between SNPs within the *SREBF1* gene and the response of fasting insulin and insulin sensitivity to a fish oil supplementation. In this study, the genetic variability within the *SREBF1* gene was associated with differences in the response of insulin and insulin sensitivity to a fish oil supplementation. *SREBF1* gene may be an important candidate to study in order to understand the discrepancies observed in the impacts of fish oil on insulin resistance. Clinical trials taking into account the genetic variability within the *SREBF1* gene and observing the impact of fish oil supplementation on insulin and insulin sensitivity are warranted. Moreover, the identification of individuals with a beneficial or adverse response to fish oil is important in order to appropriately recommend its supplementation.

## Abbreviations

CPT: Cell preparation tube
DHA: Docosahexaenoic acid
EPA: Eicosapentaenoic acid
FA: Fatty acid
*FADS*: *Fatty acid desaturase*
FFQ: Food frequency questionnaire
HOMA: Homeostasis model assessment
INAF: Institute of Nutrition and Functional Foods
*IRS2*: *Insulin receptor substrate 2*
LD: Linkage disequilibrium
MAF: Minor allele frequency
n-3: omega-3
PBMC: Peripheral blood mononuclear cell
PL: Plasma lipids
PUFA: Polyunsaturated fatty acid
QUICKI: Quantitative insulin sensitivity check index
SFA: Saturated fatty acid
SNP: Single nucleotide polymorphism
SRE: Sterol regulatory element
*SREBF1*: *Sterol regulatory element binding transcription factor 1*
Total-C: Total-cholesterol
*UCP2*: *Uncoupling protein-2*.
